# Diet and Pre-Intervention Washout Modifies the Effects of Probiotics on Gestational Diabetes Mellitus: A Comprehensive Systematic Review and Meta-Analysis of Randomized Controlled Trials

**DOI:** 10.3390/nu13093045

**Published:** 2021-08-30

**Authors:** Zubaidah Hasain, Nur Aishah Che Roos, Frhana Rahmat, Marami Mustapa, Raja Affendi Raja Ali, Norfilza Mohd Mokhtar

**Affiliations:** 1 Department of Physiology, Faculty of Medicine, Universiti Kebangsaan Malaysia, Kuala Lumpur 56000, Malaysia; zubaidah8302@gmail.com; 2 Preclinical Department, Faculty of Medicine & Defence Health, Universiti Pertahanan Nasional Malaysia, Kuala Lumpur 57000, Malaysia; frhana@upnm.edu.my (F.R.); marami@upnm.edu.my (M.M.); 3 Gastroenterology Unit, Department of Medicine, Faculty of Medicine, Universiti Kebangsaan Malaysia, Kuala Lumpur 56000, Malaysia; draffendi@ppukm.ukm.edu.my; 4 GUT Research Group, Faculty of Medicine, Universiti Kebangsaan Malaysia, Kuala Lumpur 56000, Malaysia

**Keywords:** gestational diabetes, gut microbiota, insulin resistance, probiotics, glycemic control, inflammation, oxidative stress, neonatal outcome, meta-analysis

## Abstract

Dynamic interactions among gestational diabetes mellitus (GDM), gut microbiota, inflammation, oxidative stress, and probiotics are increasingly acknowledged. This meta-analysis aimed to summarize the effects of probiotics in GDM, focusing on lifestyle intervention and pre-intervention washout, in addition to metabolic, inflammation, oxidative stress, and pregnancy outcomes. Three electronic databases (i.e., PubMed, Scopus, and CENTRAL) were searched from inception until October 2020. A meta-analysis was performed, and the effect sizes were reported as either mean differences or odds ratios with 95% confidence intervals. Altogether, 10 randomized controlled trials enrolling 594 participants were included. The meta-analysis indicated that probiotics supplementation effectively reduced fasting plasma glucose by 3.10 mg/dL, and subgroup analyses suggested that the duration of intervention, number of species, pre-intervention washout period, and dietary intervention may determine the effects of probiotics. Probiotics also reduced the level of inflammatory markers (high-sensitivity C-reactive protein, interleukin-6, tumor necrosis factor-α, and malondialdehyde), incidence of macrosomia, and newborn hospitalization. In conclusion, this meta-analysis suggests that probiotics may have positive effects on metabolic, inflammation, oxidative stress, and neonatal outcomes in women with GDM. Additionally, diet and pre-intervention washout may modify the effects of probiotics. Future studies are warranted on a larger scale to ascertain the clinical significance.

## 1. Introduction

Gestational diabetes mellitus (GDM) refers to hyperglycemia diagnosed during the second or third trimester of pregnancy [1]. The prevalence of GDM in 173 countries ranges from <1% to 28% depending on country and diagnostic criteria [2]. GDM is associated with increased risk of pregnancy outcomes, such as pre-eclampsia, congenital malformations, macrosomia, shoulder dystocia, and neonatal death [3]. Women with GDM have increased risk of cardiovascular events, and their risk of developing type 2 diabetes mellitus (T2DM) is seven-fold that of healthy women [4,5]. Moreover, the children of women with GDM are predisposed to future risk of obesity and T2DM [6]. Thus, pregnant women with GDM require optimal antenatal care for the prevention of hazardous consequences.

Currently, a healthy lifestyle recommendation (diet and exercise) is the primary approach in GDM management [3,7]. Women with GDM are recommended to consume a diet with a low glycemic index (less than 55) and limit their carbohydrate intake to 35–45% of the total energy intake [3]. Daily carbohydrate intake should be divided into three small- or medium-sized meals and two-to-four snacks [3]. Additionally, daily physical activity should be performed for approximately 30 min [8]. Studies reported that 70–85% of women diagnosed with GDM maintained glucose levels with lifestyle intervention alone [7]. However, the effectiveness of lifestyle intervention in managing GDM may be challenging, as only 16–55% of pregnant women are compliant [9,10,11]. Moreover, 13% of women with GDM require supplementary hypoglycemic agents (i.e., metformin and insulin) despite lifestyle intervention [12]. Although metformin contributes to improvement in glucose control during pregnancy, approximately 2–46% of women who receive metformin during pregnancy suffer from gastrointestinal side effects, and 6% of these women stop taking metformin due to side effects [13]. Metformin should be carefully prescribed, as significant amounts of metformin travel across the placenta, and long-term safety for the offspring of women with GDM is still uncertain [7,14]. Therefore, an additional preventive strategy that is safe, well-tolerated, and efficient in overcoming poor glycemic control in pregnant women with GDM is warranted.

Recent data from previous studies strongly support the link between altered gut microbiota (gut microbiota dysbiosis) and GDM [15,16,17]. For instance, *Sutterella*, *Bacteroides*, and *Phascolarbacterium* are positively correlated with lipopolysaccharide (LPS) biosynthesis in pregnant women with GDM [17]. The underlying mechanism may be mediated by microbial components and metabolites, particularly LPS and short-chain fatty acid (SCFA). The elevation of pathogenic microbiota and LPS, as well as reduction in SCFA, may impair gut epithelial barrier integrity and induce inflammatory reactions. These factors upregulate the expression of pro-inflammatory markers and suppress the expression of anti-inflammatory markers [18,19,20]. *Sutterella* is positively correlated with C-reactive protein levels [17]. Moreover, gut microbiota dysbiosis is linked to the overproduction of oxidative stress species (ROS), elevated lipid peroxidation and oxidative stress markers, and reduction in antioxidative markers [20,21,22]. Metabolic pathways, including the insulin signaling pathway, peroxisome proliferator-activated receptor (PPAR) signaling, and adipocytokine signaling pathway, are significantly depleted in women with GDM [15]. The perturbation of inflammation, oxidative stress reactions, and metabolic pathways is associated with insulin resistance, and this association may explain abnormal lipid and glucose metabolism in pregnant women [15,23,24]. The crosstalk between gut microbiota dysbiosis and GDM signifies an alternative preventive target in pregnant women with GDM [19].

Probiotics are defined as “live microorganisms that, when administered in adequate amounts, confer a health benefit on the host” [25]. The beneficial effects of probiotics supplementation are mediated by SCFA and are proven in various diseases, including GDM [26,27,28,29,30]. Certainly, the effects of probiotics may be influenced by various factors, including lifestyle, dietary intake, and pre-intervention washout period [31,32,33]. A high-fat and low-fiber Western diet is associated with *Bacteroides* enterotype [32]. Adults with *Bacteroides* enterotype show improvement in metabolic outcomes after *Bifidobacterium* intervention [32]. Meanwhile, the pre-intervention washout period refers to the duration in which participants are free from possible confounders (i.e., food or supplements containing probiotics or antibiotics) before an intervention [31,34]. This period is important to the elimination of the residual or carry-over effects of these cofounders and the determination of the true effects of the probiotic intervention [34].

Previous meta-analyses explored the effects of probiotic intervention on glycemic control [35,36,37,38,39,40,41,42], and limited meta-analyses analyzed lipid metabolism [39,40,43,44], inflammatory [27,44], oxidative stress [27,44], and pregnancy outcomes in pregnant women with GDM [41,43,44]. The majority of these reviews suggested that probiotic supplementation has beneficial effects on glycemic outcomes. For instance, in the study of Chen et al. [27], fasting plasma glucose (FPG) significantly decreased by 3.19 mg/dL and had a substantial heterogeneity (I^2^ = 78.8%) after probiotic supplementation. However, other glycemic control parameters, namely fasting serum insulin, homeostasis model assessment index (HOMA-IR), and quantitative insulin sensitivity check index (QUICKI), were not assessed. Through meta-analysis, Łagowska et al. [37] observed that improvement in FPG was dependent on GDM status and women with GDM showed a significant reduction in FPG compared with women without GDM (standardized mean difference (SMD), −0.46 mg/dL; 95% CI = −0.89, −0.03; *p* = 0.034). However, the reduction in FPG in this meta-analysis may not reflect the true effects of probiotics because of the high heterogeneity (I^2^ = 90.24%), which may be caused by the inclusion of studies that used synbiotics [37]. Synbiotics refer to the combinations of probiotics and substrates that are selectively utilized by host microorganisms (prebiotics) [31]. Despite that previous meta-analyses reported positive outcomes of probiotics in pregnant with GDM, most studies have failed to address the importance of lifestyle and pre-intervention washout period on probiotics supplementation.

Therefore, the primary aim of this review is to systematically review and conduct a meta-analysis of eligible randomized control trials (RCTs) investigating the effects of probiotics on glycemic outcomes in pregnant women with GDM. The secondary aims include assessing lipid, inflammatory, oxidative stress, maternal, and neonatal outcomes associated with probiotics supplementation in women with GDM. Additionally, this review aims to highlight the influences of lifestyle and pre-intervention washout on probiotic effects in order to provide a reference for future studies.

## 2. Materials and Methods

This review conforms to the Preferred Reporting Items for Systematic reviews and Meta-Analyses (PRISMA) 2020 statement [45]. The protocol to this review is registered on PROSPERO and can be accessed online (available from https://www.crd.york.ac.uk/prospero/display_record.php?ID=CRD42021226352, accessed on 17 May 2021).

### 2.1. Search Strategy

A comprehensive search was performed in the electronic databases PubMed, Scopus, and CENTRAL from inception until the fourth week of October 2020. The last search was performed on the 26th of October 2020. The following terms were used, including synonyms and closely related words as index terms or free-text words: (“pregnancy” OR “gestational“ OR “maternal diabetes” OR “gestational diabetes”) AND (“Probiotic” OR “Lactobacillus” OR “Bifidobacter”) AND (“glycemic control” OR “glucose”). Based on previous reports [27,33], the genus *Lactobacillus* and *Bifidobacter* were two commonly used probiotics as intervention for GDM; hence, they were incorporated into the search strategy. The full search strategy is detailed in Supplementary Materials Table S1. The search for relevant studies was limited to only RCTs that involved human subjects and were published in English. No geographical restriction was applied. Additionally, the references of eligible studies and relevant systematic reviews were manually screened for other eligible studies.

### 2.2. Study Criteria and Selection

Studies that met the following criteria were included: (i) The population must consist of adult pregnant women regardless of weight status (normal, overweight, or obese) diagnosed with GDM according to oral glucose tolerance test and were not on any hypoglycemic agents. No gestational age restriction was applied, even though most guidelines recommend screening for GDM between 24 and 28 weeks gestation [1]. (ii) The intervention included probiotics supplementation after GDM diagnosis, regardless of gestational age, for at least a 4-week duration. The intervention is not limited to any probiotics genus, species, or strain, as well as the number of strain and dose used. (iii) The control group must be placebo or no treatment. (iv) They must be able to report primary outcome measures: mean reduction of glycemic control biomarkers, such as FPG, HbA1c, insulin, HOMA-IR, and QUICKI. Secondary outcome measures included lipid profiles, inflammatory markers, oxidative stress markers, maternal, and neonatal outcomes. (v) Finally, the study design needed to include RCTs with at least two parallel arms comparing probiotics and control. The exclusion criteria were as follows: (i) population—pregnant women with pre-existing type 1 and/or type 2 diabetes mellitus, gastrointestinal disorders, and chronic diseases; and (ii) intervention—fermented foods (without details of bacteria), prebiotics, or synbiotics.

After deduplication, the titles and abstracts of the remaining articles were screened by two independent reviewers (N.A.C.R. and M.M.). The full texts of eligible articles were assessed, and any conflict between the reviewers was discussed until a consensus was reached. A third reviewer (Z.H.) was consulted to provide conflict resolution during each of these stages.

### 2.3. Data Extraction and Management

Data extraction into a standardized form was performed by two independent reviewers (F.R. and Z.H.). Any disagreement was resolved by consensus. Study characteristics, including geographical setting, participants’ demographic information (e.g., mean age, parity status, and gestational age), study duration, intervention, and control used, were extracted. Additionally, data related to lifestyle and pre-intervention washout period for probiotics and antibiotics were extracted. Pre-intervention washout is defined as the duration where participants were free from ingesting food and/or supplements containing probiotics and/or antibiotics. Primary outcomes (glycemic control parameters, such as FPG, insulin level, HOMA-IR, and QUICKI) and secondary outcome measures (lipid profiles, inflammatory markers, oxidative stress markers, maternal, and neonatal outcomes) were also extracted. For studies with more than two interventional arms, data from only the relevant study arms were considered. Studies by Dolatkhah et al. [46] and Hajifaraji et al. [47] were conducted in the same population at the same time and location. Hence, they were considered together in this review. The former [46] focused on the effects of probiotics on glycemic parameters, whereas the latter [47] reported the effects of probiotics on inflammatory and oxidative stress biomarkers.

In the case of missing or incomplete information, the respective author was contacted by email, and the missing data were requested if necessary.

### 2.4. Quality Assessment

The methodological quality of the included studies was assessed by using the revised Cochrane risk of bias tool for RCT (RoB 2) [48]. This tool comprises five domains, evaluating (1) random sequence generation (selection bias), and allocation concealment (selection bias), (2) blinding of participants and personnel (performance bias), (3) blinding of outcome assessment (detection bias), (4) incomplete outcome data (attrition bias), (5) selective reporting (reporting bias), and other biases. Quality assessment for each study was conducted independently by the two reviewers (N.A.C.R. and M.M.).

### 2.5. Statistical Analysis

For the meta-analysis, all data were analyzed by using the Review Manager (RevMan) 5.4 software [49]. The odds ratio (OR) was used in reporting the effect size of dichotomous data, whereas the mean difference (MD) was used in reporting the effect size of continuous data, together with their 95% confidence intervals (CIs). The heterogeneity between studies was assessed by using Higgin’s I^2^ statistic [50]. An I^2^ value that was less than 25% was regarded as low heterogeneity, whereas an I^2^ that was greater than 75% was regarded as high heterogeneity. A random-effect (RE) model was used in pooling data to account for potential heterogeneity. A *p*-value of less than 0.05 indicated statistical significance. Sensitivity analysis was conducted by the exclusion or inclusion of studies for the evaluation of the results’ robustness. A funnel plot was not reported, as the meta-analyses included less than ten studies.

## 3. Results

### 3.1. Study Selection and Study Characteristics

**Figure 1:**
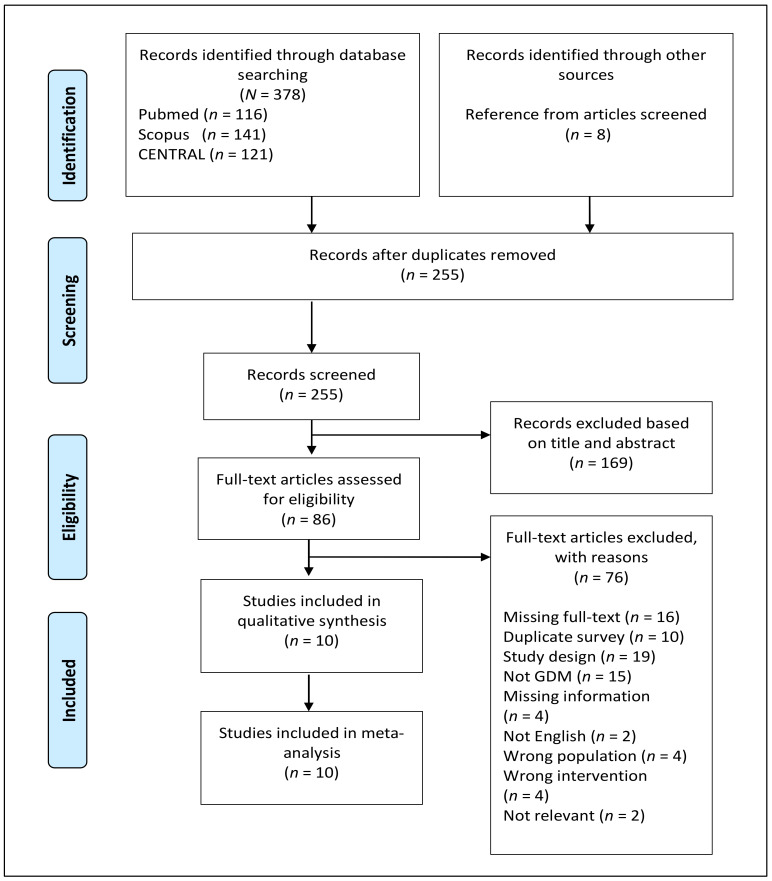
PRISMA flowchart for search strategy and study selection process.

The total of electronic search and manual screening of article references returned 386 citations (Figure 1). After deduplication, a total of 255 records were screened, and 169 were excluded because of their irrelevant titles and abstracts. The full texts of the remaining 86 records were assessed for eligibility, and only ten studies were included in the final qualitative and quantitative analyses. A total of 76 studies were excluded because of the reasons described in the study selection process (Figure 1). The general characteristics of the ten included studies are summarized in Table 1. All the included RCTs implemented a parallel-arm and double-blind design comparing probiotics with placebo. The studies were conducted in Iran [20,46,47,51,52,53,54,55], Thailand [56], and Ireland [57]. The publication date ranged from 2015 to 2019. All the participants of the included studies were assessed for GDM status with a “one-step” 2-h 75 g oral glucose tolerance test (OGTT), and only one study [57] employed a “two-step” 3-h 100 g OGTT during the second trimester onwards. The earliest assessment was conducted at 18 weeks of gestation. GDM diagnosis was performed according to the American Diabetes Association (ADA) guidelines. Meanwhile, GDM diagnosis for three studies was performed according to the International Association of Diabetes and Pregnancy Study Groups [56], Australian Diabetes in Pregnancy Society [52], and O’Sullivan’s diagnostic criteria [57]. Lindsay et al. [57] included pregnant women diagnosed with impaired glucose tolerance (IGT) and GDM. In total, 594 pregnant women with GDM and not on any hypoglycemic agents were included in the present review and meta-analysis. They were evenly and randomly divided into probiotics and placebo groups. The mean age of the participants ranged from 26.5 to 33.5 years. Six studies recruited only primigravida [20,46,47,51,54,56,57], and one study recruited primigravid and multigravid women [52]. Five studies [46,52,53,55,56] documented the pre-intervention washout period for probiotics (between 1 week and 3 months), and only two studies [46,56] reported the pre-intervention washout period for antibiotics (4 weeks).

Probiotics composition, vehicle, dose, frequency, timing of consumption, and duration of intervention varied among studies. All studies, except one [57], used multispecies probiotics, which included *Lactobacillus* and *Bifidobacterium*. Two studies [46,52] reported *Streptococcus thermophilus* as part of the probiotics composition used. The vehicles for probiotics supplementation in the studies were in capsule forms, except in one study [55], in which the probiotics were supplemented in the form of yogurt. The probiotics doses ranged from 10^6^ to 112.5 × 10^9^ colony forming units (CFU)/capsule. Probiotics supplementation was given once daily, except in one study [52], in which probiotics supplementation was given twice daily. One study [56] instructed participants to consume probiotics after a morning meal, whereas another study instructed participants to consume probiotics once daily after a meal of participant’s choice [57]. The durations of the interventions were 4–8 weeks (mean = 6.5 weeks; median = 6 weeks). An equal number of participants (*n* = 17 participants) from the probiotics and placebo groups required hypoglycemic agents during the intervention. Six studies reported excellent compliance (>90%), of which three [20,46,53] studies achieved 100% compliance to randomized treatment. All six studies measured compliance by unconsumed capsule counting. Participants were instructed to return medication containers or unused capsules, so that the remaining capsules could then be subtracted from the total number provided.

Lifestyle recommendations, assessments, and findings are summarized in Supplementary Materials Table S2. Participants were advised to undertake healthy lifestyles, particularly diet, in four studies [46,47,56,57], and five studies instructed participants to maintain their routine diets and physical activities [20,52,53,54,55]. Meanwhile, Babadi et al. [51] allowed participants to maintain their routine diets and lifestyles in addition to healthy diet recommendations. All studies, with the exception of four [46,47,51,53], have reported that participants were advised to avoid food or supplements related to probiotics. Dietary intakes were monitored with a 3-day dietary record [20,51,53,54,57] or a 24-h dietary recall [46,47,52,56]. Physical activities were assessed in six studies, measured as metabolic equivalents in hours/day [51,53,54], or were categorized into three groups (low, moderate, and high) [46,47,56] (Supplementary Materials Table S2).

### 3.2. Quality Assessment

**Figure 2:**
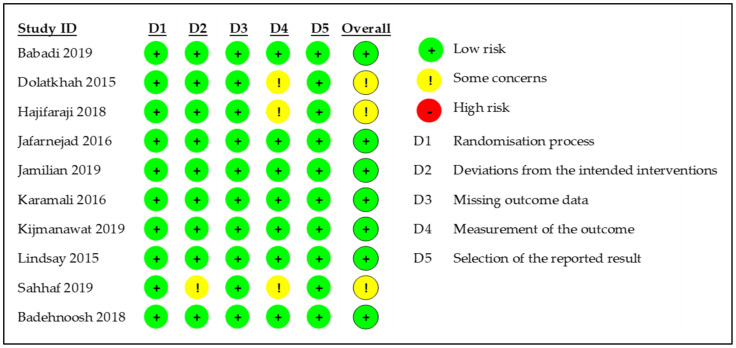
Risk of bias summary: reviewers’ judgement on the risk of bias item for included studies. Green symbol represents low risk of bias (RoB), yellow symbol represents unclear RoB, and red symbol represents high RoB.

The risk of bias assessments of the included studies is summarized in Figure 2. In general, the quality of most of the included studies is good, and seven of 10 studies had a low risk of bias. The remaining studies [46,47,55] had an unclear risk of bias. Randomization was stratified according to body mass index (BMI) and age [20,51,53,54], FPG and BMI [46,47], or age and period of amenorrhea [55]. Three studies conducted an intention-to-treat (ITT) analysis [20,54,55], six studies performed a per-protocol (PP) analysis, and one [57] study conducted ITT and PP analyses (Figure 2).

### 3.3. Effects of Probiotics on Glycemic Control

**Figure 3:**
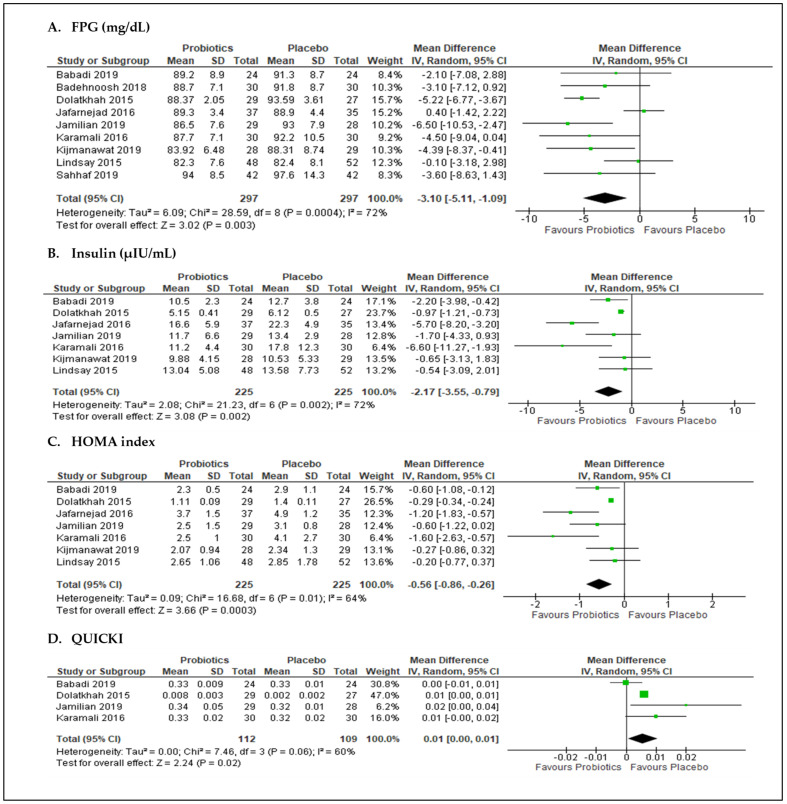
Meta-analysis of probiotics versus placebo on glycemic outcomes: (**A**) FPG, fasting plasma glucose; (**B**) insulin, (**C**) HOMA, homeostasis model assessment index; and (**D**) QUICKI, quantitative insulin sensitivity check index.

The effect of probiotics on FPG levels in GDM was reported in nine studies (Figure 3A). Of these studies, eight reported decreases in FPG levels; however, differences were statistically significant only in three studies [46,53,56]. In a random-effect meta-analysis, the pooled effect estimates from nine studies (*n* = 594 participants) resulted in statistically significant decreases in FPG levels in the probiotics groups versus placebo groups, with an MD of −3.10 mg/dL (95% CI = −5.11, −1.09; *p* = 0.003). Heterogeneity was substantial at I^2^ of 72%. For insulin parameters, the pooled results from seven studies [46,51,52,53,54,56,57] (*n* = 450 participants) resulted in significant decreases in fasting serum insulin levels and HOMA-IR in participants that received probiotics versus the placebo, which had MD values of −2.17 µIU/mL (95% CI = −3.55, −0.79; *p* = 0.002; I^2^ = 72%; Figure 3B) and −0.56 (95% CI = −0.86, −0.26; *p* = 0.0003; I^2^ = 64%; Figure 3C), respectively. However, the pooled data from four studies [46,51,53,54] (*n* = 221 participants) that reported the effects of probiotics on QUICKI showed no significant difference from the placebo (Figure 3D).

#### 3.3.1. Sensitivity Analysis

A sensitivity analysis was performed to assess the robustness of probiotics’ effects on FPG level in GDM. A comparable summary of effect size, direction, and statistical significance was obtained with a fixed-effect (FE) model meta-analysis (MD −2.97 mg/dL; 95% CI = −3.90, −2.03; *p* < 0.001). A sensitivity analysis was performed, and an additional vitamin-D arm was included as part of the control group in the trial by Jamilian et al. [53]. The addition increased the number of participants (*n* = 624 participants). Similarly, a comparable pooled effect size and magnitude were obtained with FE meta-analysis (MD −2.67 mg/dL; 95% CI = −3.60, −1.74; *p* < 0.001) and RE meta-analysis (MD −2.55 mg/dL; 95% CI = −4.45, −0.65; *p* = 0.008). A notably lower heterogeneity was observed (I^2^ = 69%). Three studies were excluded [46,51,53] in a sensitivity analysis considering only studies reporting that advice on avoidance of food and/or supplements consisting of probiotics was given to participants. The exclusion reduced the number of participants (*n* = 433 participants) and, consequently, the pooled effect size in both FE (MD −1.16 mg/dL; 95% CI = −2.43, 0.11; *p* = 0.07) and RE meta-analysis (MD −1.96 mg/dL; 95% CI = −3.94, 0.02; *p* = 0.05). A markedly lower heterogeneity was observed (I^2^ = 48%). The direction of the summary effects is comparable, albeit not statistically significant.

#### 3.3.2. Subgroup Analyses

Subgroup analyses were performed to explore the factors that may have contributed to the heterogeneity observed in the FPG level meta-analysis. Analysis based on treatment duration showed that studies conducted for 6 weeks or less yielded a pooled MD of −3.26 mg/dL (95% CI = −5.25, −1.27; *p* = 0.001), whereas studies conducted longer than 6 weeks showed a pooled MD of −2.73 mg/dL (95% CI = −7.06, 1.59; *p* = 0.22). A subgroup analysis based on the number of species that constitute the composition of probiotics used showed a pooled MD of −2.67 mg/dL (95% CI = −4.49, −0.85; *p* = 0.004) in studies that used less than four species and a pooled MD −3.28 mg/dL (95% CI = −6.94, 0.37; *p* = 0.08) in studies that used four or more species. When probiotics washout duration was considered, the pooling of studies that had a short period of washout (<2 weeks) resulted in an MD of −0.06 mg/dL (95% CI = −1.78, 1.65; *p* = 0.94). Studies that had a longer washout period had an MD of −5.27 mg/dL (95% CI = −6.63, −3.91; *p* < 0.0001). Additionally, a subgroup analysis based on dietary intervention showed a pooled FPG with MD of –3.16 mg/dL (95% CI = −5.90, −0.41; *p* = 0.02) in participants who received dietary advice from a dietician. An MD of −3.14 mg/dL (95% CI = −6.09, −0.18; *p* = 0.04) was obtained from participants who continued their usual pre-intervention diet (Table 2).

### 3.4. Effects of Probiotics on Lipid Parameters

The meta-analysis of data from four studies [51,53,54,57] (*n* = 265 participants) indicated no significant difference between probiotics supplementation versus placebo in terms of effects on total cholesterol level (MD −3.60 mg/dL; 95% CI = −16.26, 9.07; *p* = 0.58; I^2^ = 39%; Supplementary Materials Figure S1A), high-density lipoprotein cholesterol (MD −1.25 mg/dL; 95% CI = −5.48, 2.99; *p* = 0.56; I^2^ = 59%; Supplementary Materials Figure S1B), low-density lipoprotein cholesterol level (MD −6.36 mg/dL; 95% CI = −14.89, 2.16; *p* = 0.14; I^2^ = 0%; Supplementary Materials Figure S1C), and triglycerides levels (MD −10.40 mg/dL; 95% CI = −24.68, 3.89; *p* = 0.15; I^2^ = 0%; Supplementary Materials Figure S1D).

### 3.5. Effects of Probiotics on Inflammatory Biomarkers

**Figure 4:**
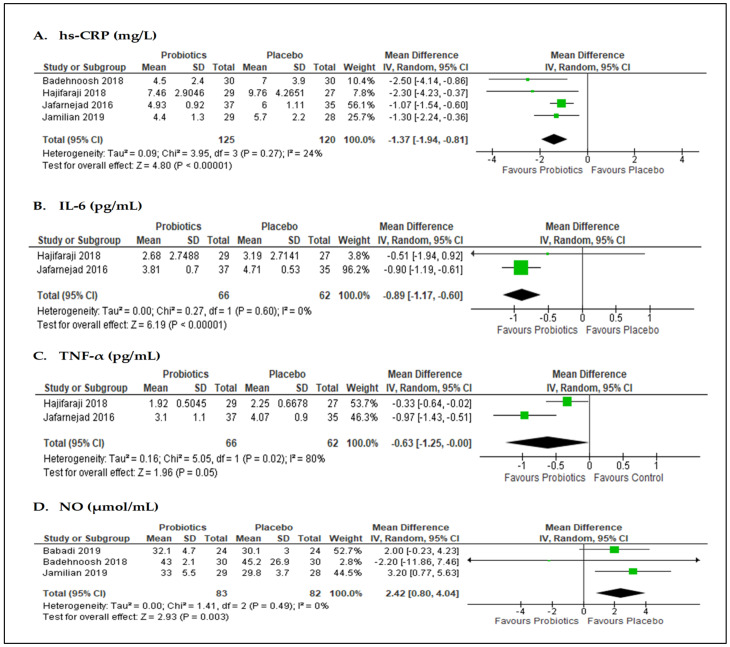
Forest plots for the meta-analysis of inflammatory markers: (**A**) hs-CRP, high-sensitivity C-reactive protein; (**B**) IL-6, interleukin; and (**C**) TNF-α, tumor necrosis factor-alpha; and (**D**) NO, nitric oxide.

The effect of probiotics on high-sensitivity C-reactive protein (hs-CRP) was reported in four studies [20,47,52,53] (*n* = 245 participants; Figure 4A). The meta-analysis of data from these studies showed that hs-CRP levels were significantly reduced in the probiotics group versus placebo, with MD of −1.37 mg/L (95% CI = −1.94, −0.81; *p* < 0.001; I^2^ = 24%). Similarly, the pooled data from two studies [47,52] (*n* = 128 participants) showed that interleukin-6 (IL-6) levels (MD −0.89 pg/mL; 95% CI = −1.17, −0.60; *p* < 0.001; I^2^ = 0%; Figure 4B) and tumor necrosis-alpha (TNF-α) levels (MD −0.63 pg/mL; 95% CI = −1.25, −0.00; *p* = 0.05; I^2^ = 80%; Figure 4C) significantly decreased in the probiotics group compared with the placebo. Three studies [20,51,53] (*n* = 165 participants) reported the effects of interventions on nitric oxide (NO) levels (Figure 4D). However, the pooled data showed that probiotics supplementation did not contribute to the reduction in NO level (MD 2.42 µmol/L; 95% CI = 0.80, 4.04; *p* = 0.003; I^2^ = 0%).

### 3.6. Effects of Probiotics on Oxidative Stress Biomarkers

**Figure 5:**
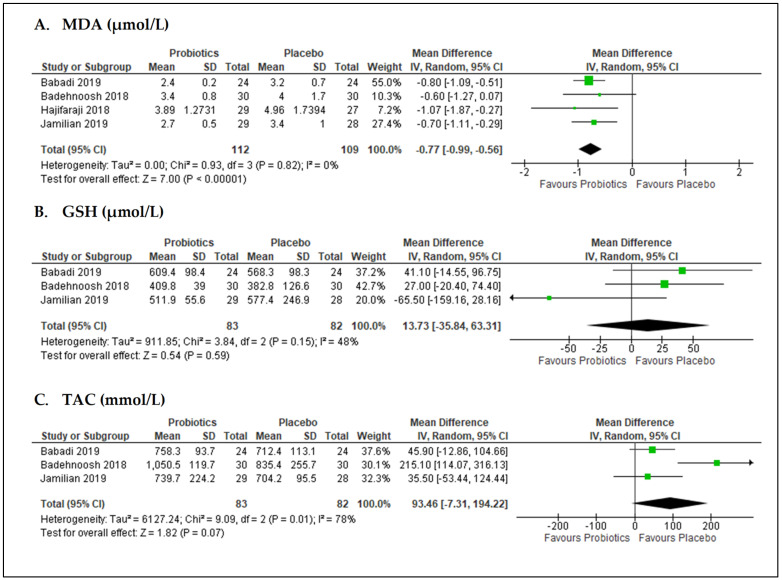
Forest plots for the meta-analysis of oxidative stress markers: (**A**) MDA, malondialdehyde; (**B**) GSH, glutathione; and (**C**) TAC, total antioxidant capacity.

Overall, four studies [20,47,51,53] (*n* = 221 participants) reported the effects of probiotics on malondialdehyde (MDA) levels (Figure 5A). The pooled data from these studies showed probiotics significantly decreased the level of MDA in comparison to placebo with MD of −0.77 µmol/L (95% CI = −0.99, −0.56; *p* < 0.001; I^2^ = 0%). No significant difference in effect was observed with probiotics versus placebo for other oxidative biomarkers. The pooled data for glutathione (GSH) and total antioxidant capacity (TAC) levels from three studies [20,51,53] (*n* = 165 participants) showed MD 13.73 µmol/L (95% CI = −35.84, 63.31; *p* = 0.59; I^2^ = 48%; Figure 5B) and MD 93.46 mmol/L (95% CI = −7.31, 194.22; *p* = 0.07; I^2^ = 78%; Figure 5C), respectively.

### 3.7. Effects of Probiotics on Maternal Outcomes

As shown in Supplementary Materials Figure S2A, three studies [20,53,57] (*n* = 217 participants) reported information on incidence of pre-eclampsia in mothers with GDM. Pooling of data from these studies indicated no significant difference in odds of developing pre-eclampsia between the probiotics versus control groups (OR 0.89; 95% CI = 0.32, 2.46; *p* = 0.82; I^2^ = 0%). Four studies [20,53,55,57] (*n* = 301 participants; Supplementary Figure Materials S2B) reported the gestational age at delivery. No significant difference in terms of period of gestation at birth was observed between the probiotics and placebo groups, with summary effects of MD 0.04 week (95% CI = −0.30, 0.38; *p* = 0.81; I^2^ = 0%). Data from four studies [20,53,54,57] (*n* = 301 participants; Supplementary Materials Figure S2C) suggested that the likelihood of cesarean delivery was slightly lower in mothers receiving probiotics that in the placebo; however, the summary effect estimate was not statistically significant (OR 0.66; 95% CI = 0.34, 1.29; *p* = 0.23; I^2^ = 39%). Similarly, no significant difference in the odds of premature birth was observed after the pooling of data from two studies [20,53] (*n* = 147 participants; Supplementary Materials Figure S2D), with OR of 1.49 (95% CI = 0.23, 9.50; *p* = 0.67; I^2^ = 0%).

### 3.8. Effects of Probiotics on Neonatal Outcomes

**Figure 6:**
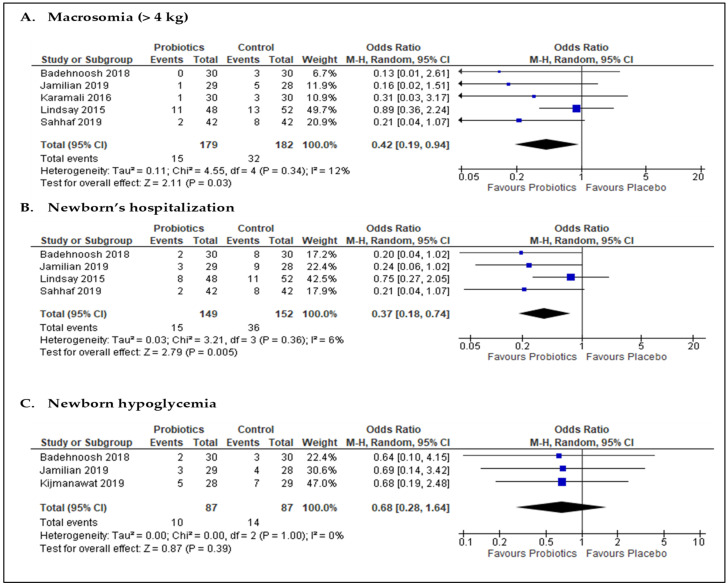
Forest plots for the meta-analysis of neonatal outcomes: (**A**) macrosomia, (**B**) newborn’s hospitalization, and (**C**) newborn hypoglycemia.

Five studies [20,53,54,55,57] (*n* = 361 participants; Figure 6A) reported the number of babies with macrosomia. The meta-analysis of data from these studies indicated the odds of macrosomia was significantly lower in the probiotics group than in the placebo group (OR 0.42; 95% CI = 0.19, 0.94; *p* = 0.03). Heterogeneity observed was low, with I^2^ of 12%. Likewise, the pooled data from four studies [20,53,55,57] (*n* = 301 participants; Figure 6B) suggested that the likelihood of newborns’ hospitalization for all reasons was significantly lower in the probiotics group than in the placebo group (OR 0.37; 95% CI = 0.18, 0.74; *p* = 0.005; I^2^ = 6%). Three studies [20,53,56] (*n* = 174 participants) reported the number of babies with hypoglycemia at birth. The pooled data from these studies resulted in an OR of 0.68 (95% CI = 0.28, 1.64; *p* = 0.39) and an I^2^ of 0%, indicating no significant difference between the two intervention groups (Figure 6C).

## 4. Discussion

The present meta-analysis indicates that probiotics supplementation has beneficial effects on metabolic outcomes, including glycemic, inflammatory, and oxidative stress parameters, together with neonatal outcomes in women with GDM. However, no significant impact was observed on either lipid or maternal outcomes.

During pregnancy, pregnant women with underlying pancreatic-β-cell dysfunction are unable to overcome metabolic, hormonal, and inflammatory changes associated with pregnancy adaptations [58,59]. The worsening of insulin resistance, hyperglycemia, and hyperlipidemia in susceptible pregnant women can be attributed in part to gut microbiota dysbiosis [15,60,61]. Gut microbiota dysbiosis in women with GDM is characterized by elevation of pathogenic microbiota, such as *Parabacteroides distasonis*, and depletion of beneficial butyrate-producing bacteria, such as *Bifidobacterium* [60]. However, the mechanism that underpins the beneficial effects of probiotics in pregnant women with GDM is still poorly understood. Supplementation with probiotics may play a role in the modulation of the gut microbial composition and may elevate the composition of beneficial butyrate-producing gut microbiota and SCFA production [19]. The interaction between SCFA and regulation of gestational glucose homeostasis is aided by a G-protein-coupled receptor (i.e., free fatty acid receptor-2) [43]. While strengthening gut epithelial permeability by the upregulation of tight junction proteins, probiotics inhibit the adhesion of pathogenic microbiota and reduce the elevation of LPS in the systemic circulation [62,63,64,65]. The elevation of SCFAs and reduction in LPS inhibit inflammatory pathways, consequently reducing the expression of pro-inflammatory markers (i.e., hs-CRP, IL-6, and TNF-α) [66,67]. Moreover, SCFA is effective in reducing lipid peroxidation and oxidative stress markers (i.e., MDA), as well as increasing antioxidant markers (i.e., TAC and GSH) [20,27]. The attenuation of inflammation and reactive oxidative stress reactions may improve insulin signaling pathways and glucose metabolism in pregnant women with GDM [19,23,68]. In addition, SCFA maintains glucose homeostasis by regulating the secretion of intestinal peptides (i.e., glucagon-like peptide-1 (GLP-1) and peptide YY (PYY)) [27]. GLP-1 helps to increase insulin signaling and delays gastric emptying [56]. Another potential marker includes the expression of the PPAR-γ gene, which is involved in the regulation of host metabolism (i.e., insulin sensitivity, lipid, and glucose) [51]. Babadi et al. [51] demonstrated that probiotics supplementation significantly increased the expression of PPAR-γ gene in GDM, indicating the possible mechanism by which probiotics affect metabolic parameters.

### 4.1. Summary of the Findings

Our meta-analyses found that probiotics supplementation resulted in statistically significant reductions in glycemic control markers in women with GDM (i.e., FPG, fasting serum insulin, and HOMA-IR) except QUICKI. Probiotics supplementation reduced FPG by 3.10 mg/dL in women that received probiotics in a random-effect meta-analysis. Although the presence of heterogeneity was substantial, the point estimates for the majority of the included studies were in the same direction favoring probiotics. Our findings on FPG corroborated the previous meta-analyses [27,35,37,38,44] that reported the effects of probiotics on glycemic outcomes in GDM. By contrast, Han et al. [43] reported no significant reduction in FPG after probiotics supplementation in women with GDM. However, they only included five studies in the meta-analysis as subgroups, and their review included healthy and GDM pregnancies [43]. Additionally, many meta-analyses investigating the effects of probiotics in GDM reported significant effects on fasting serum insulin and HOMA-IR markers [35,37,40,42,43,44], except two meta-analyses [36,41]. Improvement in fasting serum insulin and HOMA-IR indicates the potential roles of probiotics in improving insulin sensitivity [41]. Ramanathan et al. [38] reported that probiotics supplementation has favorable effects on FPG and insulin, but their analysis included pregnant women with and without GDM [38]. Meanwhile, our meta-analysis suggested that probiotics have no significant effect on lipid parameters, and our findings are in agreement with the results of previously published meta-analyses [39,40]. Data were obtained from only four RCTs, and short duration of intervention may be inadequate to elicit significant changes in lipid metabolism [31]. Nonetheless, significant reductions in total cholesterol [43] and triglycerides [43,44] were observed in other previous meta-analyses. Our results may not be comparable to these meta-analyses because they included women without GDM [43] and included synbiotics as one of the interventions in their analysis [44].

Women with GDM have higher levels of inflammatory and oxidative stress markers than women with normoglycemic pregnancies [69,70]. Low-grade inflammation and oxidative stress reactions occurring in GDM play important roles in the development of pathological insulin resistance [20]. Hence, probiotics supplementation is postulated to modulate these undesirable reactions and thereby reduces the risk of GDM complications. The present meta-analysis found inconsistent results. Probiotics showed favorable effects on certain inflammatory and oxidative stress markers (i.e., hs-CRP, IL-6, TNF-α, and MDA) but not on NO, GSH, and TAC. However, these results should be interpreted with caution because of the presence of substantial heterogeneity and the limited data. Similar inconsistencies were also reported by recently published meta-analyses [27,44]. Owing to the limited available data, deducing the association of probiotics supplementation with inflammatory and oxidative stress markers is currently impossible.

Failure to maintain nearly normal glucose levels in pregnant women with GDM is associated with an unfavorable prognosis, as it threatens the survivability of mothers and their offspring [6]. We found that the rate of newborns’ hospitalization for any reason and macrosomia were significantly lower in the probiotics group. In addition, we found that the rate of cesarean delivery was slightly lower in the probiotics groups than in the placebo group; however, the difference was not statistically significant. Nonetheless, probiotics supplementation did not show a significant effect on other pregnancy outcomes (i.e., incidence of pre-eclampsia, gestational age at delivery, premature birth, and hypoglycemia at birth). Consistently, previous meta-analyses reported no significant differences in pregnancy outcomes [41,42,43,44], except the decreased rate of premature delivery [43], decreased neonatal birth weight [41], and decreased incidence of neonatal hyperbilirubinemia [44] in the probiotics group.

### 4.2. Limitations, Strengths, and Future Directions

Overall, probiotics supplementation showed good evidence of positive outcomes in women with GDM. The majority of the clinical trials documented good compliance rates (>90%) without any side effects. However, probiotics supplementation should be cautiously prescribed, as it may be unsuitable for high-risk populations (e.g., immunocompromised patients, ill infants, and hospitalized patients) [71]. In the present analysis, we observed variabilities and conflicting findings, which may be contributed by several factors. The relatively small number of included studies and their corresponding sample sizes may have influenced the accuracy of the effect estimates generated in our meta-analysis. Furthermore, the presence of remarkable heterogeneity across studies subjected the results to be interpreted with caution. Nonetheless, the sensitivity analyses conducted showed evidence of stability in the effect estimate generated at least for the primary outcome of interest. Subgroup analyses were performed to explore heterogeneity and found that the duration of intervention, number of species used, pre-intervention washout, and dietary intervention modified the effects of probiotics on the primary outcome.

Most studies included *Bifidobacterium* and *Lactobacillus* species, but only a few had specified the probiotic strains [46,47,57]. Specifying probiotic strains is important because the evidence of probiotic effectiveness is linked to specific probiotic strains [33]. Multiple probiotic species may exert improved outcomes in terms of the normalization of gut microbial composition and increase SCFA production because of the synergistic and cooperative interactions among different probiotic species [33,72]. Accordingly, our subgroup analysis for FPG showed that probiotics intervention that applied four and more probiotic species had a better effect on FPG, consistent with earlier meta-analyses [27,43]. In comparison, Lindsay et al. [57] included only *Lactobacillus salivarius* UCC188 and did not observe significant differences between probiotic and placebo groups. Another possible reason is that they used a different diagnostic approach for participant recruitment.

The standardization of probiotic dose reporting is vital to the comparison of studies. In our meta-analysis, the probiotic doses in most trials were more than 10^9^ CFU. However, we were unable to proceed with subgroup analysis according to the probiotic doses, as the documentation of the probiotic’s doses varied among the studies. In general, probiotic doses of more than 10^6^–10^8^ CFU/g or 10^8^–10^10^ CFU/d of viable cells are considered adequate and effective [73]. However, according to the World Gastroenterology Organisation guidelines on probiotics, no exact probiotic doses can be recommended as some probiotics may have a good impact even at low doses and some may require high doses [33]. Moreover, probiotic dose sufficiency may vary depending on the impact of probiotics on certain diseases or health outcomes [33]. For instance, Sahhaf Ebrahimi et al. [55] used the lowest probiotic doses (10^6^ CFU) in yogurt form that contained only *B. lactis* and *L. acidophilus.* This trial showed a significant reduction in glucose, HbA1c, neonatal weight, and macrosomia compared with those reported by Lindsay et al., who used higher probiotics doses (10^9^) [57]. The results from Sahhaf Ebrahimi et al. [55] may be attributed to the probiotic species and the selection of yogurt as the vehicle for probiotics delivery. The study that investigated the survival of probiotics in the gut showed that probiotics survived to an extent of 23.5% ± 10.4% of the administered dose when given in fermented milk [55]. Moreover, fermented dairy products increase probiotic survivability by the buffering action of milk/milk fat and providing protection against the harsh gastrointestinal environment (i.e., acidity, bile, and enzymes) [74]. However, the selection of yogurt as the vehicle may not be convenient, as yogurt requires chilling conditions, cannot be consumed by strict vegans, and is associated with allergy and lactose intolerance [74]. A non-dairy vehicle, such as capsule, is another option that is convenient, safe, and can maintain the survivability of probiotics [74,75]. Accordingly, most trials selected capsule forms. Regarding the best time to consume probiotics, commercial literature on probiotics documented that they can be taken anytime [76]. By contrast, Tompkins et al. [76] discovered that the survival of probiotic strains depended on the time of probiotics consumption. The best time to consume probiotics is 30 min before a meal or during a meal. Probiotics that were given 30 min after a meal did not survive as much as those given before or during a meal because they might have not been able to withstand the harsh gastrointestinal environment (i.e., acidity, bile, and enzymes) [74,76]. This finding may also be the reason for Lindsay et al. [57] to obtain no significant findings because the participants were advised to consume the probiotics after a meal.

Based on the subgroup analysis, we found that clinical trials with a short duration of intervention (6 weeks and below) favored significant FPG reduction than those with long probiotics intervention as reported by a previous study [27]. The possible factor may be that RCTs that conducted long probiotics interventions are few. Han et al. [43] observed that probiotics interventions for 8 weeks and above resulted in great reductions in serum insulin and HOMA-IR. Samah et al. [77] reported a similar finding, which showed that a long period of probiotics intervention exerted a beneficial effect on cardiovascular risk factors in adults with T2DM. Moreover, International Scientific Association for Probiotics and Prebiotics consensus suggested that at least 12 weeks of intervention is required to show significant improvements in metabolic outcomes (i.e., adiposity and glycemic control) [31]. In addition, extending the duration of probiotics intervention to the postpartum period is beneficial, as women with GDM have persistent postpartum glucose intolerance and risk of developing T2DM within 3 to 4 years after delivery [78].

Pre-intervention washout period for probiotics and antibiotics is a crucial inclusion criteria upon participant recruitment. Probiotics may persist in the gut from 1 week up to 3 months [62,79,80], whereas antibiotic effects on the gut microbiota may persist from 6 weeks up to 6 months after discontinuation [81,82]. Recommendation on the ideal pre-intervention washout duration for probiotics and antibiotics may depend on probiotics species, antibiotic type, dose, duration of consumption, probiotics survivability in situ, and characteristics of the study subjects [31]. In general, a minimum of 2–4 weeks washout period for probiotics is sufficient to remove residual/carry-over effects [83,84]. However, a longer washout period (i.e., more than 4 weeks) should be considered for individuals with slow transit time [31,85]. Meanwhile, the washout period for antibiotics should be at least 4 weeks after discontinuation [31]. We found great improvement in FPG with 0% heterogeneity in the trials that included participants with long probiotics pre-intervention washout period. However, limited RCTs documented the pre-intervention washout period, and only one RCT considered a long pre-intervention washout period (3 months) for probiotics [53]. Therefore, future trials should focus on probiotics and antibiotics pre-intervention washout period to eliminate potential confounding factors.

Lifestyle (diet and physical activity) is a potential confounding factor, as it is associated with the modulation of the gut microbiota [32,86,87,88,89,90]. Diet should be monitored, as food containing onions and wheat may contribute a source of prebiotic substrates and fermented food may consist of live microorganisms [25,31]. To exclude confounding factors, most studies recommended their participants to maintain regular lifestyles. However, the documentation of lifestyle changes pre- and post-intervention was incomplete and absent in certain trials. The trials only instructed participants to avoid food and supplements containing probiotics. A review by Facchinetti et al. [91] suggested that a combination of lifestyle and probiotics interventions demonstrated better outcomes than probiotics intervention alone. As expected, our subgroup analysis showed that studies that provided dietary advice to their participants showed slightly more reduction in the FPG than the studies that instructed their participants to maintain a similar pattern of diet throughout the intervention.

The results obtained from the present meta-analysis may not represent the global population, as a majority of RCTs included were conducted in Iran. Differences in genetic makeup and environmental factors, including culture and dietary patterns in different populations, may influence gut microbiota profile, hence determining the effects of probiotics [32,87,92]. Furthermore, the evaluation of gut microbiota profile and its associated biomarkers, including SCFAs, LPSs, GLP-1, PYY, PPAR-γ, and HbA1c, is lacking. Regular HbA1c measurement is required, as it is part of the ADA recommendation, and this marker signifies the average glucose levels for 12 weeks [1]. Thus, a long multiple-strain probiotics intervention from different geographical locations, accompanied with the analysis of gut microbiota profile, associated biomarkers (i.e., SCFAs, LPSs, GLP-1, PYY, PPAR-γ, and HbA1c), and multi-omics approaches (i.e., proteomics, transcriptomics, and metabolomics), can be a future novel approach. In addition, the assessment of all related parameters after probiotics discontinuation may support and confirm the effects of probiotics supplementation. Moreover, evaluation of gut microbiota profile pre- and post-intervention may be useful to determine the level of probiotics compliance among participants.

Despite these limitations, the present meta-analysis has several strengths that are noteworthy. First, this review focused strictly on probiotics intervention and their effects on pregnant women with GDM. Second, we only included RCTs with highly reliable evidence of the effectiveness of interventions, and most of the included RCTs had a low risk of bias. Third, in comparison with the earlier published meta-analysis, the present study included additional RCTs that were not available previously, hence resulting in the improvement of the precision of effect estimates. Finally, the present review highlighted the lifestyle and pre-intervention washout period as important factors that possibly influence the effects of probiotics.

## 5. Conclusions

This meta-analysis suggests that probiotics supplementation may offer positive effects on glycemic, inflammatory, and neonatal outcomes in pregnant women with GDM. Moreover, dietary intervention and pre-intervention washout are potential modifiers of probiotics’ effects. Nevertheless, the findings should be interpreted with caution because of the remarkable heterogeneity across studies. Further investigation is worthwhile, and future studies considering variables that were discussed in this review and elucidating the benefits of probiotics in women with GDM are recommended.

## Figures and Tables

**Table 1:** Characteristics of randomized control trials included in this meta-analysis.

First Author	Study Design	Sample Size		Study Population			Washout Period	Probiotics Intervention		Requirement of Hypoglycemic Agents during Intervention	Compliance (%) Side Effects	Outcomes Measured
Year, Reference	Country Dx of GDM	Total		Gravida Status	Age (Years)		Probiotics Antibiotics	Probiotic Species	Vehicle Dosage (CFU) Frequency Duration			
		I	C	Gestational Age	I	C						
Babadi 2019, [51]	P, RCT, DB Iran 2-h 75 g OGTT	48 24 24		Primigravida 24–28 weeks	28.3 ± 4.3	29.0 ± 4.2	NR NR	*B. bifidum* *L. acidophilus* *L. casei* *L. fermentum*	Capsule 2 × 10^9^ CFU/g each Once daily 6 weeks	No	>90% No	Genetic Glycemic Lipid Inflammatory Oxidative stress Weight gain
Badehnoosh 2018, [20]	P, RCT, DB Iran 2-h 75 g OGTT	60 30 30		Primigravida 24–28 weeks	28.8 ± 5.4	27.8 ± 3.7	NR NR	*B. bifidum* *L. acidophilus* *L. casei*	Capsule 2 × 10^9^ CFU/g each Once daily 6 weeks	Yes—5 women (3-I and 2-C)	100% NR	Inflammatory Glycemic Oxidative stress Pregnancy ^3^
Dolatkhah ^1^ 2015, [46] Hajifaraji ^2^ 2018, [47]	P, RCT, DB Iran 2-h 75 g OGTT	56 29 27		Primigravida 24–28 weeks	28.1 ± 6.2	26.5 ± 5.2	2 weeks 4 weeks	*B.* BB-12 *L. acidophilus* LA-5 *L. delbrueckii* Bulgaricus LBY-27 *Streptococcus thermophilus* STY-31	Capsule 4 biocap > 4 × 10^9^ CFU Once daily 8 weeks	Yes—4 women (2-I and 2-C)	100% No	Glycemic Inflammatory Oxidative stress Weight gain
Jafarnejad 2016, [52]	P, RCT, DB Iran 2-h 75 g OGTT	72 37 35		Primigravida, Multigravida ~26 weeks	32.4 ± 3.1	31.9 ± 4.0	10 days NR	*B. breve, B. longum* *B. infantis, L. acidophilus* *L. plantarum, L. paracasei L. delbrueckii* subsp. Bulgaricus *Streptococcus thermophilus*	VSL#3 capsule 112.5 × 10^9^ CFU/capsule Twice daily 8 weeks	Yes—7 women (2-I and 5-C)	NR No	Glycemic Inflammatory
Jamilian 2019,[53]	P, RCT, DB Iran 2-h 75 g OGTT	57 29 28		Primigravida 24–28 weeks	31.2 ± 5.9	29.9 ± 3.7	3 months NR	*B. bifidum* *L. acidophilus* *L. casei* *L. fermentum*	Capsule 8 × 10^9^ CFU/g Once daily 6 weeks	Yes—3 women (1-I and 2-C)	100% NR	Glycemic Lipid Inflammatory Oxidative stres Pregnancy ^3^
Karamali 2016, [54]	P, RCT, DB Iran 2-h 75 g OGTT	60 30 30		Primigravida 24–28 weeks	31.8 ± 6.0	29.7 ± 4.0	NR NR	*B. bifidum* *L. acidophilus* *L. casei*	Capsule 2 × 10^9^ CFU/g each Once daily 6 weeks	No	>90% No	Glycemic Lipid Weight gain
Kijmanawat 2019, [56]	P, RCT, DB Thailand 2-h 75 g OGTT	57 28 29		Primigravida 24–28 weeks	32.5 ± 5.0	30.7 ± 5.1	2 weeks 4 weeks	*B. bifidum* *L. acidophilus*	Capsule 1 × 10^9^ CFU/each Once daily after meal 4 weeks	No	>90% No	Glycemic Weight gain Neonatal
Lindsay 2015, [57]	P, RCT, DB Ireland 3-h 100 g OGTT	100 48 52		Primigravida 18–34 weeks Included both IGT and GDM	33.5 ± 5.0	32.6 ± 4.5	NR NR	*L. salivarius* UCC118	Capsule 100 mg at 10^9^ CFU Once daily after meal 4–6 weeks	Yes—15 women (9-I and 6-C)	NR NR	Glycemic Lipid Pregnancy ^3^
Sahhaf Ebrahimi 2019, [55]	P, RCT, DB Iran 2-h 75 g OGTT	84 42 42		NR 24–28 weeks	31.6 ± 6.0	31.6 ± 5.5	1 week NR	*B. lactis* *L. acidophilus*	Yogurt 300 g/day (10^6^ CFU) Once daily 8 weeks	No	NR NR	Glycemic Neonatal

Dx, diagnosis; GDM, Gestational diabetes mellitus; OGTT, oral glucose tolerance test; IGT, impaired glucose tolerance; I, intervention/probiotics group; C, control/placebo group; CFU, colony forming unit; P, parallel; RCT, randomized control trial; DB, double-blind; NR, not reported; *B., Bifidobacterium; L., Lactobacillus*. ^1,2^ Both articles used the same population but published different outcomes. ^3^Pregnancy (maternal and neonatal outcomes).

**Table 2:** Subgroup meta-analysis of the effect of probiotics on fasting plasma glucose.

Analysis	No.	References	Random-Effects Model		Fixed-Effect Model		Heterogeneity	
			MD (95% CI)	*p*	MD (95% CI)	*p*	I^2^	*p*
Subgroup 1: Duration of intervention	9							
≤6 weeks	6	[20,51,53,54,56,57]	−3.26 [−5.25, −1.27]	0.001	−3.10 [−4.72, −1.49]	0.002	32%	0.2
>6 weeks	3	[46,52,55]	−2.73 [−7.06, 1.59]	0.22	−2.90 [−4.05, −1.75]	<0.001	91%	<0.001
Subgroup 2: Number of species	9							
≤3 species	5	[20,54,55,56,57]	−2.67 [−4.49, −0.85]	0.004	−2.64 [−4.41, −0.86]	0.004	5%	0.38
>3 species	4	[46,51,52,53]	−3.28 [−6.94, 0.37]	0.08	−3.10 [−4.20, −1.99]	<0.001	88%	<0.001
Subgroup 3: Probiotics washout period	5							
<2 weeks	2	[52,55]	−0.06 [−1.78, 1.65]	0.94	−0.06 [−1.78, 1.65]	0.94	53%	0.14
≥2 weeks	3	[46,53,56]	−5.27 [−6.63, −3.91]	<0.001	−5.27 [−6.63, −3.91]	<0.001	0%	0.56
Subgroup 4: Dietary intervention	9							
Received dietary advice	4	[46,51,56,57]	−3.16 [−5.90, −0.41]	0.02	−4.07 [−5.33, −2.80]	<0.001	67%	0.03
Maintain regular diet	5	[20,52,53,54,55]	−3.14 [−6.09, −0.18]	0.04	−1.63 [−3.02, −0.23]	0.02	69%	0.004

No., number; MD, mean difference; CI, confidence interval; *p*, *p*-value; *p*-value < 0.05 was considered statistically significant.

## Data Availability

Data are contained within the article or are available from the individual studies that were referenced throughout the text.

## Supplementary Materials

The following are available online at https://www.mdpi.com/article/10.3390/nu13093045/s1, Table S1: Search Strategy. Table S2: Lifestyle recommendations, monitoring, and findings in pregnant women with GDM. Figure S1: Forest plots for the meta-analysis of lipid parameters. Figure S2: Forest plots for the meta-analysis of maternal outcomes.
