# Supplementary material for: Diet and Pre-Intervention Washout Modifies the Effects of Probiotics on Gestational Diabetes Mellitus: A Comprehensive Systematic Review and Meta-Analysis of Randomized Controlled Trials
Source: Nutrients. 2021 Aug 30;13(9):3045. doi: 10.3390/nu13093045 (PMC8465224; doi:10.3390/nu13093045)
Supplement: Supplementary file 1 [file nutrients-13-03045-s001.zip › Supplementary Figure S1.pdf]

A. Total cholesterol (mg/dL)

Study or Subgroup	Probiotics Mean	Probiotics SD	Probiotics Total	Placebo Mean	Placebo SD	Placebo Total	Weight	Mean Difference IV, Random, 95% CI
Babadi 2018	201.7	38.8	24	214	43.1	24	20.4%	-12.30 [-35.50, 10.90]
Jamilian 2019	219.6	37.4	29	225.8	33	28	27.5%	-6.20 [-24.49, 12.09]
Karamali 2016	198.3	47.5	30	213.7	46.5	30	19.7%	-15.40 [-39.19, 8.39]
Lindsay 2015	252.5	37.12	48	241.3	42.54	52	32.5%	11.20 [-4.42, 26.82]
**Total (95% CI)**			**131**			**134**	**100.0%**	**-3.60 [-16.26, 9.07]**

Heterogeneity: Tau² = 65.00; Chi² = 4.92, df = 3 (P = 0.18); I² = 39%
Test for overall effect: Z = 0.56 (P = 0.58)

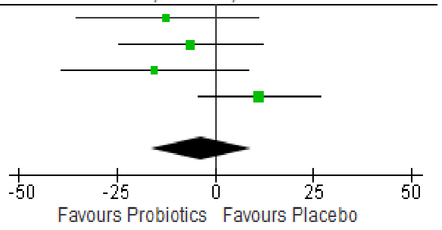

B. HDL cholesterol (mg/dL)

Study or Subgroup	Probiotics Mean	Probiotics SD	Probiotics Total	Placebo Mean	Placebo SD	Placebo Total	Weight	Mean Difference IV, Random, 95% CI
Babadi 2018	54.2	9.3	24	51.7	5.7	24	29.4%	2.50 [-1.86, 6.86]
Jamilian 2019	54.1	10.9	29	54.5	12.8	28	22.4%	-0.40 [-6.58, 5.78]
Karamali 2016	49.9	10.2	30	57.4	12.7	30	23.6%	-7.50 [-13.33, -1.67]
Lindsay 2015	64.9	14.7	48	65.4	13.5	52	24.7%	-0.50 [-6.05, 5.05]
**Total (95% CI)**			**131**			**134**	**100.0%**	**-1.25 [-5.48, 2.99]**

Heterogeneity: Tau² = 10.92; Chi² = 7.30, df = 3 (P = 0.06); I² = 59%
Test for overall effect: Z = 0.58 (P = 0.56)

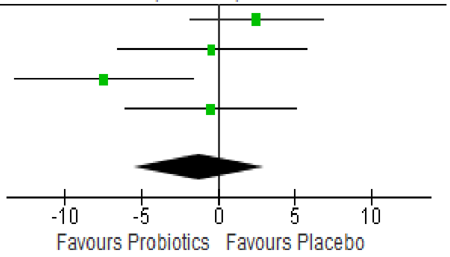

C. LDL cholesterol (mg/dL)

Study or Subgroup	Probiotics Mean	Probiotics SD	Probiotics Total	Placebo Mean	Placebo SD	Placebo Total	Weight	Mean Difference IV, Random, 95% CI
Babadi 2018	108.2	35.5	24	120.7	36.1	24	17.7%	-12.50 [-32.76, 7.76]
Jamilian 2019	134.9	34.1	29	136.9	33.4	28	23.7%	-2.00 [-19.52, 15.52]
Karamali 2016	110.2	37.7	30	113.4	33.9	30	22.1%	-3.20 [-21.34, 14.94]
Lindsay 2015	137.28	34.03	48	145.4	37.9	52	36.6%	-8.12 [-22.22, 5.98]
**Total (95% CI)**			**131**			**134**	**100.0%**	**-6.36 [-14.89, 2.16]**

Heterogeneity: Tau² = 0.00; Chi² = 0.77, df = 3 (P = 0.86); I² = 0%
Test for overall effect: Z = 1.46 (P = 0.14)

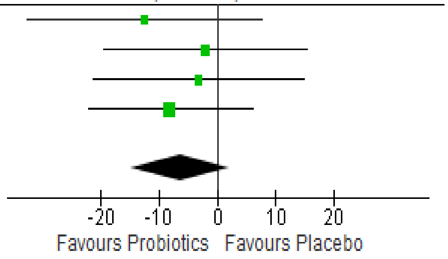

D. TG (mg/dL)

Study or Subgroup	Probiotics Mean	Probiotics SD	Probiotics Total	Placebo Mean	Placebo SD	Placebo Total	Weight	Mean Difference IV, Random, 95% CI
Babadi 2018	196.5	43.5	24	208.2	69.4	24	19.0%	-11.70 [-44.47, 21.07]
Jamilian 2019	153.1	60.5	29	171.9	42.6	28	27.8%	-18.80 [-45.89, 8.29]
Karamali 2016	191.1	71.2	30	214.8	73.7	30	15.2%	-23.70 [-60.37, 12.97]
Lindsay 2015	252.4	36.6	48	250.7	76.2	52	38.0%	1.70 [-21.45, 24.85]
**Total (95% CI)**			**131**			**134**	**100.0%**	**-10.40 [-24.68, 3.89]**

Heterogeneity: Tau² = 0.00; Chi² = 1.93, df = 3 (P = 0.59); I² = 0%
Test for overall effect: Z = 1.43 (P = 0.15)

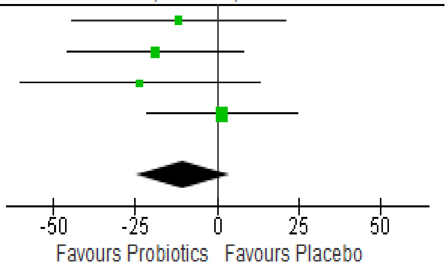

Supplementary Figure S1. Forest plots for the meta-analysis of lipid parameters: (**A**) Total cholesterol; (**B**) HDL, high density lipoprotein cholesterol; (**C**) LDL, low density lipoprotein cholesterol; and (**D**) TG, triglyceride cholesterol.
