# Supplementary material for: Diet and Pre-Intervention Washout Modifies the Effects of Probiotics on Gestational Diabetes Mellitus: A Comprehensive Systematic Review and Meta-Analysis of Randomized Controlled Trials
Source: Nutrients. 2021 Aug 30;13(9):3045. doi: 10.3390/nu13093045 (PMC8465224; doi:10.3390/nu13093045)
Supplement: Supplementary file 1 [file nutrients-13-03045-s001.zip › Supplementary Figure S2.pdf]

A. Pre-eclampsia

Study or Subgroup	Probiotics Events	Probiotics Total	Control Events	Control Total	Weight	Odds Ratio M-H, Random, 95% CI
Badehnoosh 2018	2	30	2	30	25.2%	1.00 [0.13, 7.60]
Jamilian 2019	3	29	5	28	43.9%	0.53 [0.11, 2.47]
Lindsay 2015	3	48	2	52	30.9%	1.67 [0.27, 10.43]
**Total (95% CI)**		**107**		**110**	**100.0%**	**0.89 [0.32, 2.46]**
Total events	8		9			

Heterogeneity: Tau² = 0.00; Chi² = 0.90, df = 2 (P = 0.64); I² = 0%
Test for overall effect: Z = 0.23 (P = 0.82)

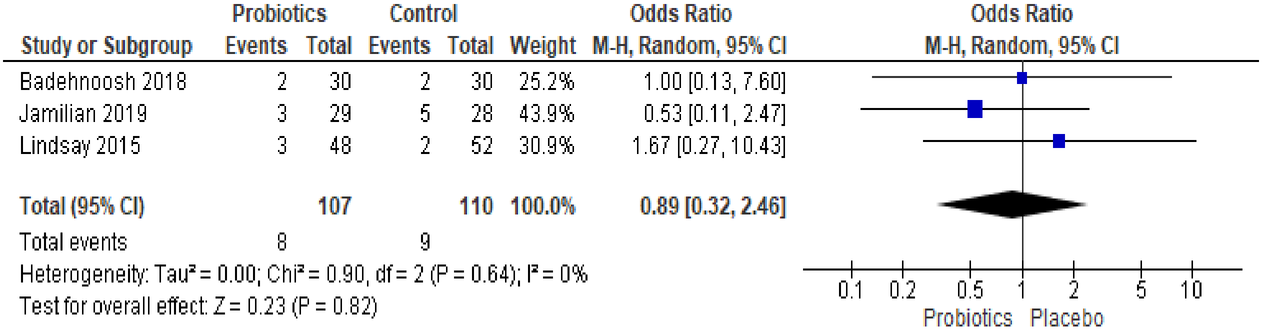

B. Gestational age at delivery (week)

Study or Subgroup	Probiotics Mean	Probiotics SD	Probiotics Total	Control Mean	Control SD	Control Total	Weight	Mean Difference IV, Random, 95% CI
Badehnoosh 2018	39.1	2.5	30	39.1	1.1	30	12.3%	0.00 [-0.98, 0.98]
Jamilian 2019	38.9	2.5	29	38.6	1.1	28	11.8%	0.30 [-0.70, 1.30]
Lindsay 2015	40.1	1.23	48	39.9	1.2	52	51.6%	0.20 [-0.28, 0.68]
Sahhaf 2019	37.7	1.9	42	38.1	1.3	42	24.2%	-0.40 [-1.10, 0.30]
**Total (95% CI)**			**149**			**152**	**100.0%**	**0.04 [-0.30, 0.38]**

Heterogeneity: Tau² = 0.00; Chi² = 2.23, df = 3 (P = 0.53); I² = 0%
Test for overall effect: Z = 0.24 (P = 0.81)

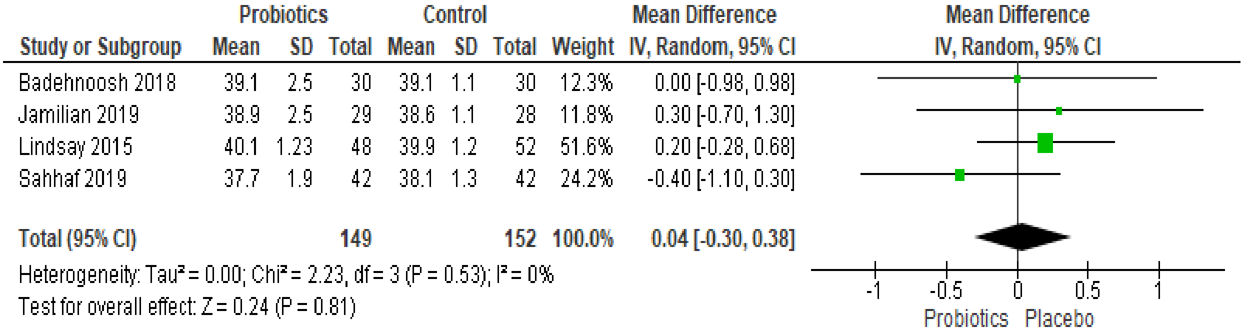

C. Cesarean section

Study or Subgroup	Probiotics Events	Probiotics Total	Control Events	Control Total	Weight	Odds Ratio M-H, Random, 95% CI
Badehnoosh 2018	6	30	14	30	22.0%	0.29 [0.09, 0.90]
Jamilian 2019	10	29	12	28	24.0%	0.70 [0.24, 2.05]
Karamali 2016	7	30	11	30	22.5%	0.53 [0.17, 1.62]
Lindsay 2015	17	48	15	52	31.5%	1.35 [0.58, 3.14]
**Total (95% CI)**		**137**		**140**	**100.0%**	**0.66 [0.34, 1.29]**
Total events	40		52			

Heterogeneity: Tau² = 0.18; Chi² = 4.94, df = 3 (P = 0.18); I² = 39%
Test for overall effect: Z = 1.21 (P = 0.23)

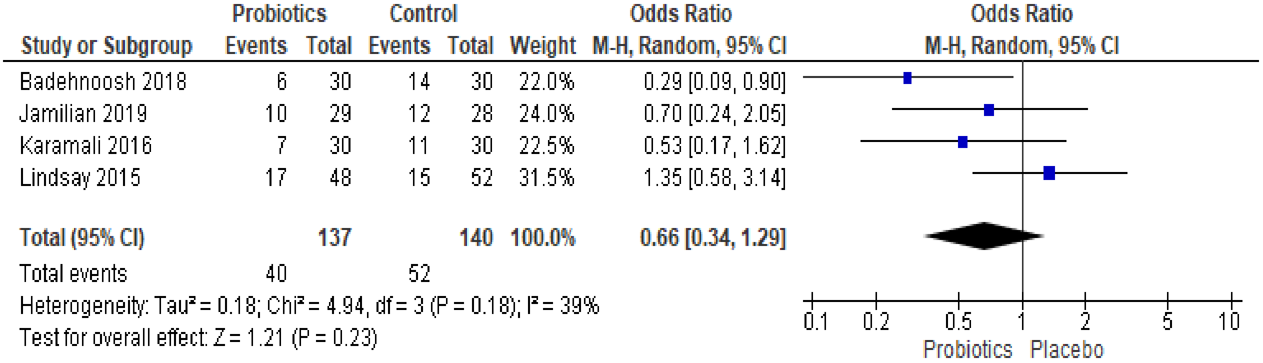

D. Preterm delivery

Study or Subgroup	Probiotics Events	Probiotics Total	Control Events	Control Total	Weight	Odds Ratio M-H, Random, 95% CI
Badehnoosh 2018	2	30	1	30	56.9%	2.07 [0.18, 24.15]
Jamilian 2019	1	29	1	28	43.1%	0.96 [0.06, 16.21]
**Total (95% CI)**		**59**		**58**	**100.0%**	**1.49 [0.23, 9.50]**
Total events	3		2			

Heterogeneity: Tau² = 0.00; Chi² = 0.16, df = 1 (P = 0.69); I² = 0%
Test for overall effect: Z = 0.42 (P = 0.67)

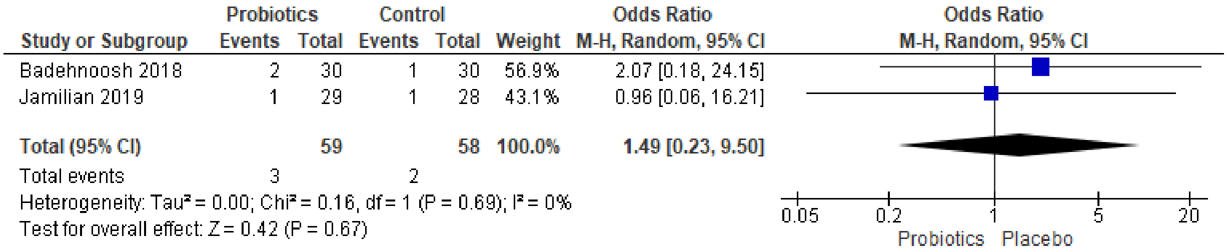

Supplementary Figure S2. Forest plots for the meta-analyses of maternal outcomes: (**A**) Pre-eclampsia; (**B**) Gestational age at delivery; (**C**) Caesarian section; and (D) Preterm delivery.
