# Supplementary material for: Diet and Pre-Intervention Washout Modifies the Effects of Probiotics on Gestational Diabetes Mellitus: A Comprehensive Systematic Review and Meta-Analysis of Randomized Controlled Trials
Source: Nutrients. 2021 Aug 30;13(9):3045. doi: 10.3390/nu13093045 (PMC8465224; doi:10.3390/nu13093045)
Supplement: Supplementary file 1 [file nutrients-13-03045-s001.zip › Supplementary Table S1.pdf]

Supplementary Table 1. Search Strategy

Database: SCOPUS (26 October 2020)

	Keywords	Search result
1.	Pregnancy OR Gestation* OR "Matern* diabetes" OR "Gestation* diabetes"	956,491
2.	Probiotic* OR Lactobacill* OR Bifidobacter*	83,475
3.	"glyc?mic control" OR glucose OR insulin OR HbA1c OR HOMA	1,067,614
4.	#1 AND #2 AND #3	141
	October (fourth week)	

Database: PubMed (26 October 2020)

	Keywords	Search result
1.	Pregnancy OR Gestation* OR "Matern* diabetes" OR "Gestation* diabetes"	1,067,050
2.	Probiotic* OR Lactobacill* OR Bifidobacter*	65,776
3.	"glyc?mic control" OR glucose OR insulin OR HbA1c OR HOMA	834,949
4.	#1 AND #2 AND #3	116
	October (fourth week)	

Database: Cochrane (26 October 2020)

	Keywords	Search result
1.	Pregnancy OR Gestation* OR "Matern* diabetes" OR "Gestation* diabetes"	66145
2.	Probiotic* OR Lactobacill* OR Bifidobacter*	9304
3.	"glyc?mic control" OR glucose OR insulin OR HbA1c OR HOMA	91849
4.	#1 AND #2 AND #3	121
	October (fourth week)	

Total search result: 378

After duplicates removed: 256
