# Supplementary material for: Diet and Pre-Intervention Washout Modifies the Effects of Probiotics on Gestational Diabetes Mellitus: A Comprehensive Systematic Review and Meta-Analysis of Randomized Controlled Trials
Source: Nutrients. 2021 Aug 30;13(9):3045. doi: 10.3390/nu13093045 (PMC8465224; doi:10.3390/nu13093045)
Supplement: Supplementary file 1 [file nutrients-13-03045-s001.zip › Supplementary Table S2.pdf]

Supplementary Table S2. Lifestyle recommendations, monitoring, and findings in pregnant women with GDM.

First Author<br>Year, (ref)	Received Healthy Lifestyle Recommendations	Advised to Avoid Foods or Supplements Consist of Probiotics	Advised to Maintain Similar Routine Diet and Physical Activity during Intervention	Methods of Dietary Assessment and Findings	Methods of Physical Activity Assessment and Findings
Babadi, 2019, [51]	Yes	NR	Yes	3-day diet record at 0, 3, and 6 WOI. Documented no significant changes. (Data not shown).	Measured as metabolic equivalents (METs) in hours/day. Findings: NR.
Badehnoosh 2018, [20]	NR	Yes	Yes	3-day diet record at 1, 3, and 5 WOI. No significant changes.	NR
Dolatkhah[a] 2015, [46]<br>Hajifaraji[b] 2018, [47]	Yes	NR	NR	24-h diet recall for 3 consecutive days at 0, 4, and 8 WOI. Findings: NR	Baseline PA was categorized into three groups (low, moderate, and high). Mostly have a low level of physical activity (70%).
Jafarnejad 2016, [52]	NR	Yes	Yes	24-h recall for 3 days at 0 and 8 WOI. No significant changes.	NR
Jamilian 2019,[53]	NR	NR	Yes	3-day diet record at 0, 3, and 6 WOI. No significant changes.	Measured as METs at 0, 3, and 6 WOI. No significant changes.
Karamali 2016, [54]	NR	Yes	Yes	3-day diet record at 0, 3, and 6 WOI. No significant changes	Measured as METs as hours/day at 0, 3, and 6 WOI. No significant changes.
Kijmanawat 2019, [56]	Yes	Yes	NR	Baseline 24-h diet recall for three consecutive days after 2 WOI. Dietary intake was similar between the two groups.	Baseline PA was categorized into three groups (low, moderate, and high) based on 3-day PA. Mostly have a low level of physical activity (>78%).
Lindsay 2015, [57]	Yes	Yes	NR	3-day diet record for three consecutive days after 2 WOI. Dietary findings NR	NR
Sahhaf Ebrahimi 2019, [55]	No	Yes	Yes	NR	NR

NR, not reported; WOI, weeks of intervention; MET, metabolic equivalent; PA, physical activity.
